# Potential Antipsychotic Side Effects and Adverse Events of Assisted Living Residents With Dementia

**DOI:** 10.1093/geroni/igaa057.2484

**Published:** 2020-12-16

**Authors:** Anna Beeber, Sheryl Zimmerman, Christopher Wretman, Stephanie Miller, Kush Patel, Philip Sloane

**Affiliations:** 1 University of North Carolina at Chapel Hill, Chapel Hill, North Carolina, United States; 2 Cecil G. Sheps Center for Health Services Research, Chapel Hill, North Carolina, United States; 3 Northeast Ohio Medical University, Rootstown, Ohio, United States

## Abstract

This presentation provides the findings from a descriptive study examining the potential adverse events and side effects of antipsychotic medications prescribed to assisted living (AL) residents with dementia drawn from interviews with family members and chart review on 238 AL residents, from 91 AL communities in seven US states. We found that 85% of family reported that medication had been administered for agitation or aggression, 93% of the sample experienced at least one potential side effect, and 19% experienced five or more. The most common potential side effects were neurologic/psychological effects (89% of residents), and somnolence during the day (81%). Six percent of the sample experienced at least one potential adverse event. This work implies a need for caution when prescribing antipsychotics to older adults with dementia in AL. Medication management efforts should extend to monitoring AL residents for potential side effects and adverse events from specific psychoactive medications.

